# A Surgical Method to Improve the Homeostasis of CSF for the Treatment of Alzheimer’s Disease

**DOI:** 10.3389/fnagi.2016.00261

**Published:** 2016-11-02

**Authors:** Yang Qin, Jian W. Gu

**Affiliations:** ^1^Department of Neurosurgery, Chengdu Military General HospitalChengdu, China; ^2^Department of Neurosurgery, The 306th Hospital of PLABeijing, China

**Keywords:** cerebrospinal fluid, amyloid-β, Alzheimer’s disease, homeostasis, ventriculo-peritoneal shunting

## Abstract

Reduced cerebrospinal fluid (CSF) production and increased resistance to CSF outflow are considered to be associated with aging, and are also characteristics of Alzheimer’s disease (AD). These changes probably result in a decrease in the efficiency of the mechanism by which CSF removes toxic molecules such as amyloid-β (Aβ) and tau from the interstitial fluid space. Soluble Aβ is potently neurotoxic and dysfunctional in CSF circulation and can accelerate the progression of AD. Current therapies for AD exhibit poor efficiency; therefore, a surgical method to improve the homeostasis of CSF is worthy of investigation. To achieve this, we conceived a novel device, which consists of a ventriculo-peritoneal shunt, an injection port and a portable infusion pump. Artificial CSF (ACSF) is pumped into the ventricles and the ACSF composition, infusion modes and pressure threshold of shunting can be adjusted according to the intracranial pressure and CSF contents. We hypothesize that this active treatment for CSF circulation dysfunction will significantly retard the progression of AD.

## Introduction

Alzheimer’s disease (AD) is an age-related dementia that represents a serious social problem in the aging population worldwide (Kandimalla et al., 2011, 2014; Marešová et al., 2015). This chronic degenerative disease of the brain is characterized clinically by progressive deterioration of memory and other cognitive domains, along with profound changes in personality and behavior (Dubois et al., 2015). Amyloid-β (Aβ) and protein tau have long been recognized as the major pathogenic factors (Kandimalla et al., 2013), and are highlighted in the major consensus criteria for the diagnosis of AD at autopsy (Mirra et al., 1991). The exact etiology of AD is unclear, but it is presumed to include factors such as age, genetics, inflammation, head trauma, etc. (Castellani et al., 2010). Currently, cholinesterase inhibitors and memantine are the only treatments that were widely confirmed to be marginally beneficial for AD patients (Schmidt et al., 2015). Although not yet fully confirmed, other protocols that are expected to be beneficial include: increasing the cerebral blood flow, which is believed to be able to delay and even improve the clinical presentation of AD (Goldsmith, 2014); nerve growth factor, which can prevent neuronal degeneration (Tuszynski, 2007); antioxidants (Zandi et al., 2004); statins, which may decrease cerebral Aβ (Barone et al., 2014); non-steroidal anti-inflammatory drugs, which may lower Aβ production (McGeer and McGeer, 2013); hormone replacement therapy, in which estrogen enhances cerebral blood flow, preventing cholinergic neuron atrophy, reducing oxidative stress, and modulating the effects of nerve growth factors (Goutte et al., 2002); blocking of excitotoxicity, which has been approved for treating the advanced stages of AD (Kurz and Grimmer, 2014); a Mediterranean diet (Singh et al., 2014); the Aβ vaccine, which removes excess Aβ from the brain by activating specific T cell responses (Gilman et al., 2005); immunotherapy, which is used to reduce the Aβ load in the brain (Paquet et al., 2015) and induce the secretion of effectors, which inhibit the protease that initiates cleavage of Aβ protein precursor leading to the production of Aβ (Chang et al., 2007); and neural stem cells transplantation, which may be capable of replacing lost or damaged cells and reverse the course of AD (Zhang et al., 2013). Most of these treatments were evaluated in Aβ-based therapeutic trials, which indicated that Aβ is a breakthrough point in the development of cure for AD. However, so far, the results have not met with these high expectations (Iqbal et al., 2014). As the therapeutic based on decreasing amyloid plaque remains suboptimal and phosphorylated tau plays an important role in the pathological process of AD, the therapy was moving from Aβ to tau (Giacobini and Gold, 2013).

The physiological functions of cerebrospinal fluid (CSF), which is the internal environment of the brain, include amortization, acid-base buffering and transport of electrolytes, molecules and micronutrients (Spector et al., 2015). Normal CSF circulation is important to ensure homeostasis of the internal environment of the brain. Unfortunately, with aging, CSF production decreases and outflow resistance increases (May et al., 1990). In the elderly, the CSF synthesized by choroid plexus may decrease as much as 50% (Serot et al., 2003). These changes may disrupt CSF homeostasis and delay clearance of toxic molecules such as Aβ from the interstitial fluid space. Although diffuse amyloid plaques that are typically composed of Aβ are pathognomonic for AD, they are unlikely to be potently neurotoxic (Chaudhury et al., 2003). Conversely, soluble Aβ is highly neurotoxic, with effects ranging from the induction of cell death to disruption of normal neuronal function (Nichols et al., 2015). A recent study suggested that, during the very early stages of AD, Aβ in CSF may significantly increase (Maia et al., 2015). With the progression of AD, CSF Aβ has decreased and pronounced Aβ has deposited in the choroid plexus and arachnoid granulations, where the CSF is produced and absorbed (Kalaria et al., 1996). Aβ deposition exacerbates the dysfunction in CSF circulation in a vicious cycle that eventually triggers, or at least contributes to, the development of AD (Rubenstein, 1998).

To our knowledge, no animal experiments have confirmed that it is beneficial to AD by directly promoting the circulation of CSF. However, caffeine, which can increase production of CSF, was proven to be beneficial for Alzheimer animals and patients (Han et al., 2009; Cao et al., 2011; Kromhout et al., 2014). In a clinical study evaluating the effect of low-flow CSF drainage in the treatment of AD, a trend in favor of the treated group was observed, although the effect did not reach the level of statistical significance (Silverberg et al., 2002). We speculate that the reason why no better results were obtained may be due to the fact that CSF drainage improved the outflow resistance but did not effectively reinstate homeostasis of the CSF. According to the current hypothesis, a more aggressive therapy was conceived to recover the homeostasis and retard the progression of AD.

## Materials and Methods

**Figure 1:**
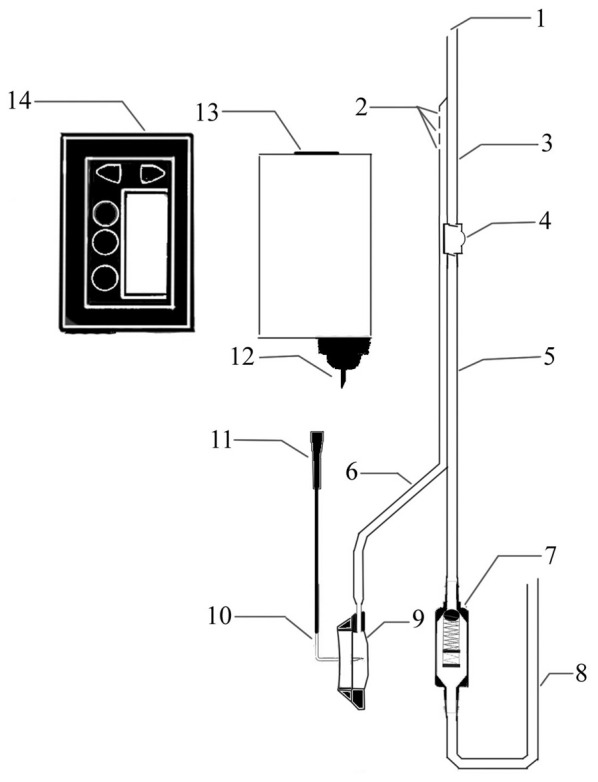
**Structure diagram of the cerebrospinal fluid (CSF) circulatory assistance device.** 1. CSF outlet; 2. Artificial CSF (ACSF) inlet; 3. Intraventricular double lumen catheter; 4. A one-way liquid storage bag; 5. Subcutaneous double lumen catheter; 6. Transfusion connecting tube; 7. Pressure control valve; 8. Peritoneal cavity catheter; 9. Infusion seat; 10. L-shaped nondestructive needle; 11. Needle connecting head; 12. Fluid needle; 13. Pre-packaged ACFS; 14. Infusion pump.

A novel device was designed to improve CSF circulation. The device consists of three main parts: an adjustable pressure ventriculo-peritoneal shunt, an injection port and portable infusion pump. The shunt and the port are connected and form a double-lumen tube to enter the ventricles of the brain. The pump, which is connected to the injection port by an infusion apparatus with an L type non-coring needle, can accommodate 150 ml artificial CSF (ACSF) and accurately control the infusion process (Figure 1).

**Figure 2:**
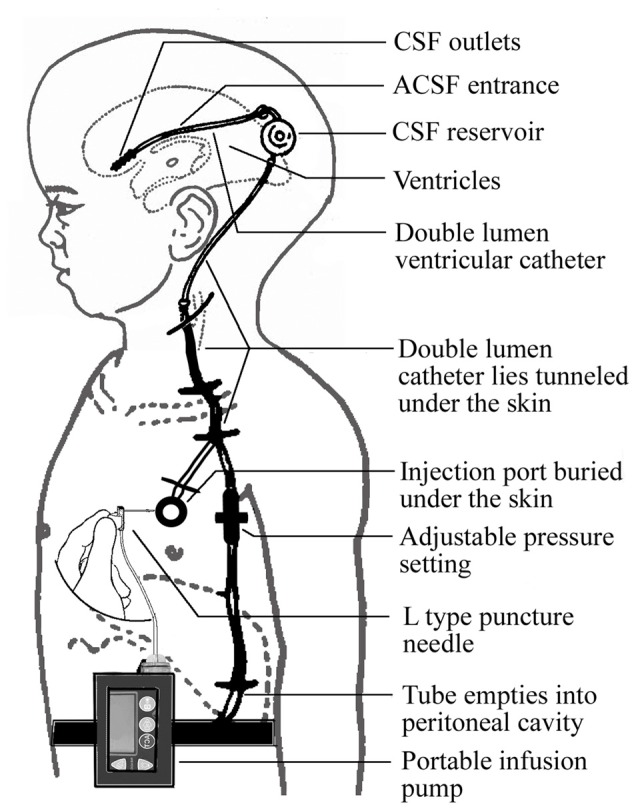
**Schematic diagram of the CSF circulatory assistance device**.

The required implantation of the ventriculo-peritoneal shunt and the injection port is performed under general anesthesia using a surgical procedure similar to that used for shunt implantation described previously (Silverberg et al., 2002). The proximal and the distal ends of the catheter are placed into the frontal horn of the lateral ventricle and the peritoneal cavity, respectively. The infusion port is implanted under the skin of the chest. The portable infusion pump can be carried by the patient (Figure 2).

The main operation mode of the device allows injection of the ACSF into the lateral ventricles to supplement the insufficient CSF, as well as drainage of the supernumerary CSF through the shunt tube. An infusion bottle containing 150 ml ACSF, which is approximately equivalent to the total amount of CSF in normal adults, is placed in the infusion pump. The ACSF is pumped continuously and slowly into the ventricles over a period of 8–12 h during the daytime. The amount of the ACSF is adjusted by measuring biological markers in the CSF such as Aβ and tau, with the aim of restoring the composition of the CSF. The difference between the intracranial pressure and the valve pressure is set at 0–20 cm H_2_O to minimize the drainage rate and maximize the CSF turnover efficiency. For patients accompanied by normal pressure hydrocephalus (NPH), the difference can be adjusted to 20–40 cm H_2_O.

## Discussion

The hypothesis that dysfunctional CSF circulation is involved in the pathogenesis of AD presents a novel therapeutic target for this disorder. Here, we describe a device that was designed for the treatment of AD according to this hypothesis. The therapy promotes CSF circulation by direct intervention in all stages of the circulation: production, turnover and clearance. CSF production in patients with AD insignificantly decreased to approximately 300 ml per day; about half the amount of normal people (Silverberg et al., 2001). In a normal adult with a total CSF volume of 150 ml, the CSF turnover rate is about four times per day. In patients with AD, reduced CSF production and enlarged ventricles cause a reduction in the turnover rate to less than 1.5 times per day. Normally, the clearance of Aβ and other neurotoxic macromolecules from the CSF is quite rapid (Ghersi-Egea et al., 1996). This process requires the continuous formation of fresh CSF to drain the macromolecular solutes in the interstitial fluid from the Virchow Robin space down concentration gradients into the subarachnoid space and from there, into the bloodstream (Nakada, 2015). The clearance is severely reduced in AD patients due to the dysfunction in CSF production and turnover, as well as the disappearance of the capillary receptors that transport Aβ from the CSF into blood (Mackic et al., 1998). Although the present treatment cannot restore normal physiological CSF circulation directly, especially not improve the production of intercellular fluid, which is driven by aquaporin-4 and very important for the clearance of Aβ and other toxins (Nakada, 2014); it can play a similar role in other form to improve CSF circulation. Briefly, the insufficient CSF production can be compensated by direct injection. By adjusting the injection speed and shunt pressure difference, the drainage volume and speed can be controlled to allow sufficient time and space for the turnover between CSF and ACSF. The result is the increase of the solute concentration difference between the CSF and the interstitial fluid, which may facilitate the free diffusion and dilution of neurotoxic substances so as to achieve the purpose of clearance. In the drainage phase, the Aβ that is not cleared by the receptor can also be discharged through the drainage tube. It needs to be pointed out that the manuscript has no intention to emphasize Aβ and ignore other potential factors. What we have done is to take Aβ as an example to illustrate the importance of CSF homeostasis for AD.

Previous studies have shown numerous similarities between AD and idiopathic NPH, including age of onset, clinical symptoms and Aβ accumulation (Graff-Radford, 2014; Martín-Láez et al., 2015). Clinical benefits have been demonstrated in a clinical trial using a form of ventriculo-peritoneal shunting, which is usually used in the treatment of NPH, for the treatment of AD, although the effects were not statistically significant (Silverberg et al., 2002). Hence we designed a more aggressive treatment plan for AD. However, not all the AD patients may benefit from this treatment. The goal of the therapeutic strategy is to delay the progression of AD rather than to cure it. The present treatment may not be suitable for the dementia stage patients whose large number of neurons have died or deteriorated. We hypothesized that it is suitable for the patients at the stage of mild cognitive impairment or preclinical stage, when the CSF composition has changed. Of course, the patient’s tolerogenic capability to anesthesia and surgery must also be considered important. The adverse events associated with the treatment may be similar to those reported for ventriculo-peritoneal shunting, especially catheter infection or obstruction; and it remains to be established in clinical trials.

The treatment hypothesis is based on the existing theory and limited indirect animal experiments and clinical trials. It is still at most preliminary. First of all, it needs to be supported by the results of direct animal experiment, which has not been clearly designed so far. Furthermore, a fully developed device that can be used in clinical trials is also not yet available. Although there is no technical barrier to the production of the equipment in its current form, financial investment is urgently needed to complete the work. We expect the preliminary idea would provide a novel view of the treatment of AD.

## Author Contributions

YQ and JWG: conception and design. YQ: completed the mamuscript. JWG: reviewed submitted version of the manuscript.

## Funding

This work was supported by the Youth Medical Innovation Research Foundation of Sichuan Province, China (Grant No. Q14007) and National Natural Science Foundation of China (Grant No. 81271395).

## Conflict of Interest Statement

The authors declare that the research was conducted in the absence of any commercial or financial relationships that could be construed as a potential conflict of interest.
